# Likelihood-of-harm/help of microsurgery compared to radiosurgery in large vestibular schwannoma

**DOI:** 10.1007/s11060-024-04732-0

**Published:** 2024-06-29

**Authors:** Sophie Shih-Yüng Wang, Gerhard Horstmann, Albertus van Eck, Marcos Tatagiba, Georgios Naros

**Affiliations:** 1 Department of Neurosurgery, Eberhard Karls University, Hoppe-Seyler-Strasse 3, Tubingen, Germany; 2 Gamma Knife Center Krefeld, Krefeld, Germany

**Keywords:** Vestibular schwannoma, Acoustic neuroma, Stereotactic radiosurgery, Microsurgery

## Abstract

**Purpose:**

It has been shown that in large vestibular schwannomas (VS), radiosurgery (*SRS*) is inferior with respect to tumor control compared to microsurgical resection (*SURGERY*). However, *SURGERY* poses a significantly higher risk of facial-function deterioration (FFD). The aim of this study was to illustrate the effectiveness in terms of number-needed-to-treat/operate (NNO), number-needed-to-harm (NNH), and likelihood-of-harm/help (LHH) by comparing both treatment modalities in large VS.

**Methods:**

This was a retrospective, dual-center cohort study. Tumor size was classified by Hannover Classification. Absolute risk reduction and risk increase were used to derive additional estimates of treatment effectiveness, namely NNO and NNH. LHH was then calculated by a quotient of NNH/NNO to illustrate the risk–benefit-ratio of *SURGERY*.

**Results:**

Four hundred and forty–nine patients treated met the inclusion criteria. The incidence of tumor recurrence was significantly higher in *SRS* (14%), compared to *SURGERY* (3%) resulting in ARR of 11% and NNO of 10. At the same time, *SURGERY* was related to a significant risk of FFD resulting in an NNH of 12. Overall, the LHH calculated at 1.20 was favored *SURGERY,* especially in patients under the age of 40 years (LHH = 2.40), cystic VS (LHH = 4.33), and Hannover T3a (LHH = 1.83) and T3b (LHH = 1.80).

**Conclusions:**

Due to a poorer response of large VS to *SRS*, *SURGERY* is superior with respect to tumor control. One tumor recurrence can be prevented, when 10 patients are treated by *SURGERY* instead of *SRS*. Thus, LHH portrays the benefit of *SURGERY* in large VS even when taking raised FFD into account.

**Supplementary Information:**

The online version contains supplementary material available at 10.1007/s11060-024-04732-0.

## Introduction

In vestibular schwannoma (VS) – a benign intracranial neoplasm located in the cerebellopontine angle (CPA), [1, 2] both, stereotactic radiosurgery (*SRS*) and microsurgical tumor resection (*SURGERY*) are valid options for choice of treatment [3–5]. Postinterventional facial functional deterioration (FFD), after *SURGERY* is especially pronounced in large VS, while *SRS* faired significantly better in respect to facial preservation [5]. However, it has previously been shown that long-term tumor control in large VS (Hannover T3-T4 / Koos III-IV) is significantly inferior in *SRS* compared to *SURGERY* [5]. This illustrates the particular challenges in clinical decision-making of large VS.

The ambivalence of treatment efficacy (reduction of tumor recurrence/progression) of *SURGERY* in light of its increased adverse effects (e.g. FFD) compared to *SRS*, needs to be illustrated in a well-rounded manner in order to translate clinical research results into clinical practice and hereby enable satisfactory patient consultation. Absolute risk reduction (ARR), absolute risk increase (ARI), and odds ratio are extensively used parameters to illustrate the benefit or disadvantage of one treatment over another [6]. However, in the context of clinical decision-making, it is also meaningful to use the measure of number needed to treat (NNT) [7, 8]. NNT is defined as the number of people needed to receive *SURGERY* instead of *SRS* to prevent an outcome over a defined time period. It has been widely used in scientific literature to communicate benefits of a treatment (e.g. medication or vaccination) and is used as an epidemiological measure for reporting treatment impact [9–12]. At the same time, treatment toxicity is reported by the equivalent number needed to harm (NNH). For risk–benefit analysis, the Likelihood-of-harm/help (LHH), calculated as the ratio of NNH to NNT, is able to illustrate trade-offs between harms and benefits of two treatments [8, 11].

However, this kind of measurement has not yet been translated to Neuro-Oncology yet. The largest branch of clinical neurooncological research focuses on high-grade glioma with its devastating prognosis and treatment comparative effectiveness analysis involving treatment toxicities remain in the background [13]. In light of benignancy of VS, the debate on different treatment modalities in VS management remains multi-faceted. The aim of this study was to illustrate the effectiveness in tumor control, to identify parameters that may indicate the effectiveness of either *SURGERY* or *SRS* in the therapy of large VS and to characterize operative benefits in terms of NNT, NNH and LHH by comparing both treatment modalities.

## Methods

### Study design and patient cohort

This was a retrospective dual-center cohort study. Study reporting followed the Strengthening the Reporting of Observational Studies in Epidemiology (STROBE) guidelines. Patients were identified by a prospectively kept registry. Previously treated VS, VS associated with Neurofibromatosis, patients with pre-interventional FFD, and small VS (Koos I-II) were excluded from this study. Data were then retrospectively collected between 2005 und 2011 from two tertiary and specialized centers involved in the treatment of VS for patients.

### Data collection

Tumor size was classified by Hannover Classification [14, 15]. Clinical state was reported by House and Brackmann (H&B) [16] and Gardner-Robertson (G&R) scale (with H&B and G&R 1–2 considered to be good outcome) [17]. Recurrence-free-survival (RFS) was assessed radiographically by gadolinium-enhanced magnetic resonance imaging (MRI) [18, 19]. The criteria for tumor recurrence/progression was progredient growth in contrast-enhanced MRI (radiographic tumor control, RTC). To exclude the described phenomenon of pseudoprogression after *SRS*, patients with tumor volume (TV) increase 6 months after SRS with stable TV afterwards or TV decrease were not graded as VS recurrence/progression [20]. The TV was measured using slice-by-slice manual contouring. In case of *SURGERY*, extent of resection (EOR) was classified by first post-operative MRI (3 months postoperative): residual contrast-enhancing tumor was defined as subtotal resection (STR), whereas gross total resection (GTR) was defined as lack of contrast-enhancement in MRI. Due to the low number and for statistical purposes, the patients with subtotal resections were excluded. The local ethics committee approved this analysis, which was conducted according to the ethical standards laid down in the Declaration of Helsinki for research involving human subjects.

### Treatment modalities

Patients treated by *SURGERY* were all operated on via the retrosigmoid approach using intraoperative electrophysiological monitoring in semi-sitting position under continuous echocardiography monitoring [21, 22]. All VS patients in the *SRS* cohort received Gamma-Knife-Radiosurgery (GKR – Elekta AB, Stockholm, Sweden) with a prescription dose of 13 Gy to the 65% isodose line [23].

### Statistical analysis

Statistical analysis was performed in R Studio (Version 1.2) using descriptive statistics. Incidence of recurrence per patient-time was calculated as the following: quotient of number of recurrent events to number of patient days. This result was then shown per 1 million days. ARR was calculated as the difference between incidence rate of recurrence or a postoperative FFD in patients treated with *SRS* and *SURGERY*. The ARR was then used to derive an estimate of treatment effectiveness, which was the NNT, defined as 1 / ARR. If NNT is negative in undesirable outcomes (e.g., the occurrence of posttreatment long-term FFD), it is usually referred to as NNH. Concerning the occurrence of a recurrence, there was a positive NNT; thus, we refer to it as number needed to operate (NNO) throughout the manuscript. In contrast, *SURGERY* was a significant predictor for the occurrence of posttreatment FFD, resulting in a negative 1/ARR relationship (i.e., NNH). LHH was then calculated by a quotient of NNH / NNO to illustrate the risk–benefit ratio [10, 11]. As the less invasive treatment option, *SRS* was used as the standard therapy in all NNO, NNH, and LHH analyses, to which *SURGERY* was then compared by considering treatment benefits and harms.

To compare nonnumeric parameters of both groups, the chi-square test was applied. For numeric parameters, Welch’s two sample t-test was used. RFS was estimated using the Kaplan–Meier method and compared between cases and controls using a log-rank test. The length of follow-up for RFS was calculated from the date of surgical or radiosurgical intervention to the date of either recurrence or the last clinical visit. Significance was defined as the probability of a two-sided type 1 error being < 5% (*p* < *0.05*). Data is presented as mean ± standard deviation (SD) if not indicated otherwise.

## Results

**Fig. 1:**
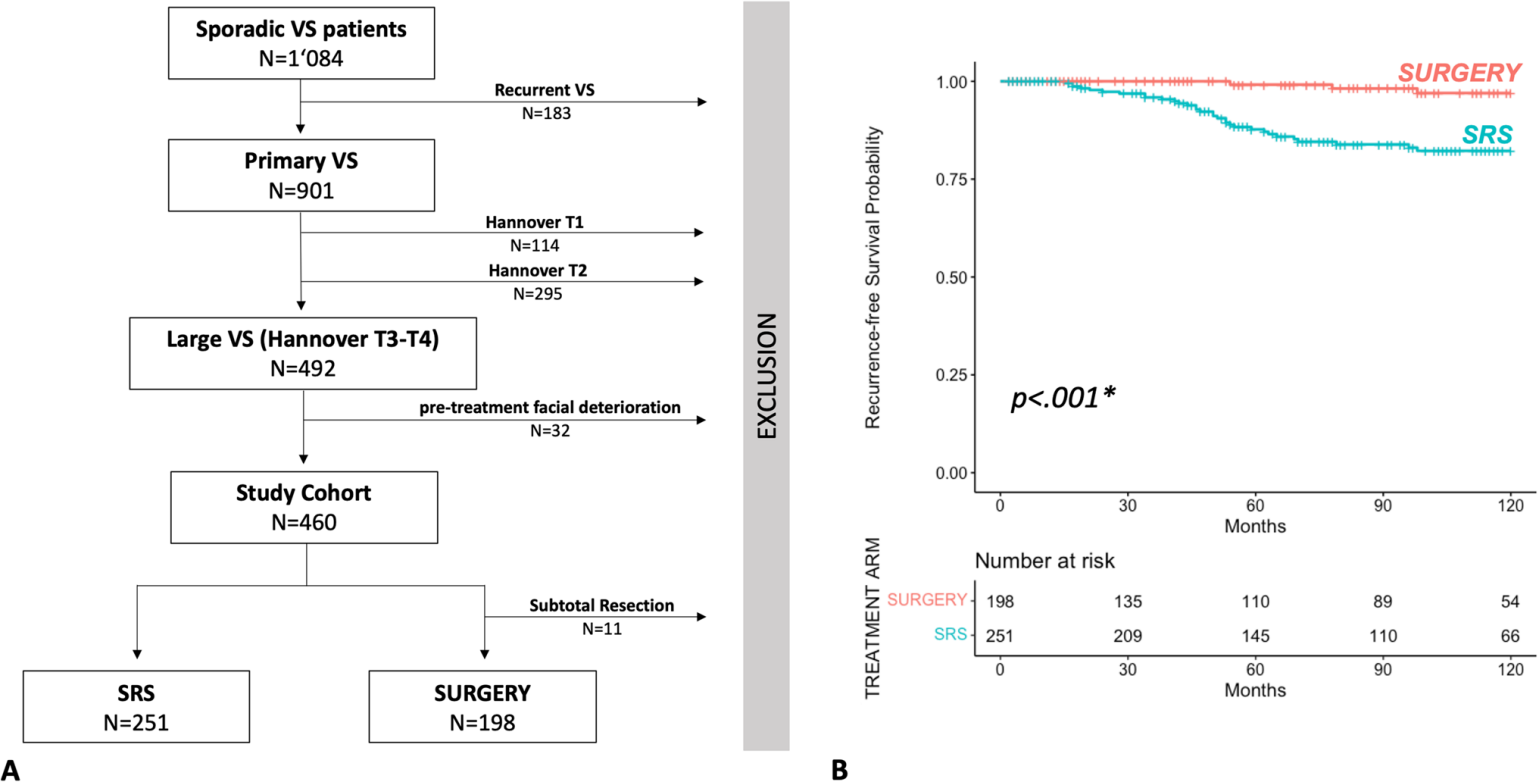
**A** Flowchart of patient cohort. **B** 10-Year Kaplan–Meier-Analysis for tumor-recurrence *SRS* versus *SURGERY*

From 2005 to 2011, 901 patients with primary and solitary VS were treated in both centers. Of those patients, *n* = 492 (55%) were classified as large VS (Hannover T3-T4) according to the Hannover-Classification and used as the main study cohort in this analysis. Patients with pre-operative FFD at HB > 1 (*n* = 31; 6%) were excluded due to the study design. From this study cohort (*n* = 460), *n* = 209 (45%) received *SURGERY*, while *n* = 251 (55%) received *SRS*. GTR was achieved in 95% (*n* = 198), while the rate for STR was at 5% (*n* = 11) with six subtotal and five near-total resection (for detailed analysis of STR subgroup, see supplementary material). The patient cohort flowchart is shown in Fig. 1A below. Mean patient age was significantly higher in the *SRS* subgroup compared to *SURGERY* (*p* < *0.001*). Cystic morphology was more often present in the surgically treated (*SURGERY*) with *p* = *0.002*. Tumor size was unequally distributed (see Table 1) with larger tumors more likely to be treated with *SURGERY* then *SRS*.

**Table 1:** Patient demographics, tumor characteristics, incidence of shunt-dependency, recurrence, clinical presentation pre- and posttreatment, treatment complications and Clavien-dindo classification (CDC)

	*All (N* = *449)*	*SRS (N* = *251)*	*SURGERY (N* = *198)*	*p-value*
*Age*	53.7 (± 13.7)	58.8 (± 12.7)	47.4 (± 12.1)	< .001*
*Female*	248 (55)	143 (57)	105 (53)	.445
*Cystic Morphology*	36 (8)	11 (4)	25 (13)	.002*
*Hannover Klassification*				
*Hannover T3*	311 (69)	193 (77)	118 (60)	< .001*
*Hannover T4*	138 (31)	58 (23)	80 (40)	< .001*
*Shunt-Dependency*	10 (2)	7 (3)	3 (2)	.523
*Recurrence*	41 (9)	35 (14)	6 (3)	< .001*
*Preoperative clinical status*				
*Good Facial function (HB1-2)*	449 (100)	251 (100)	198 (100)	1
*Functional Hearing (G&R 1–2)*	221 (49)	102 (41)	119 (60)	< .001*
*Facial Spasm*	0 (0)	0 (0)	0 (0)	1
*Tinnitus*	324 (72)	183 (73)	141 (71)	.751
*Trigeminus*	64 (14)	32 (13)	32 (16)	.342
*Vertigo*	272 (61)	157 (63)	115 (58)	.382
*Postoperative clinical status*				
*Good Facial function (HB1-2) last FU*	430 (96)	250 (99)	180 (91)	< .001*
*Functional Hearing (G&R 1–2) last FU*	112 (25)	67 (27)	45 (23)	.379
*Facial Spasm*	13 (3)	13 (5)	0 (0)	< .001*
*Tinnitus*	206 (46)	169 (67)	37 (19)	< .001*
*Trigeminus*	35 (8)	27 (11)	8 (4)	.008*
*Vertigo*	173 (39)	126 (50)	47 (24)	< .001*
*Treatment Complications / Side Effects*	30 (7)	6 (2)	24 (12)	< .001*
*CDC*				
*2*	10 (2)	5 (2)	5 (3)	.752
*3a*	14 (3)	0 (0)	14 (7)	< .001*
*3b*	6 (1)	1 (1)	5 (3)	.092
> *4*	0 (0)	0 (0)	0 (0)	1

Values are presented as the number of patients (%) unless indicated otherwise. Significant *p*-values (< 0.05) are highlighted with *

Preinterventional clinical parameters were similar in both groups. The rate of functional hearing at last follow-up was similar in both groups with 27% in *SRS* and 23% in *SURGERY (p* = *0.625)*. Tinnitus, trigeminal symptoms, and vertigo were significantly improved by S*URGERY* (Table 1). New-onset facial spasm was an *SRS*–specific event with an incidence of 5% in *SRS*. In the *SRS* cohort, 0.3% experienced a FFD. Of all patients treated with *SURGERY*, 30% experienced a relevant early postinterventional FFD (H&B > 2). However, of these patients, 69% improved after 1 year and at last follow-up (H&B < 2). Therefore, the rate of permanent FFD (H&B > 2) at last follow-up was 9% in *SURGERY*.

The rate of direct postoperative FFD (H&B > 2) was 22% in patients under 40 years old, 34% in patients 40–50 years old, 37% in patients 50–60 years old, and 29% in patients older than 60 years. Notably, 54% of patients under age 40 with a poor facial outcome (H&B 3–6) directly after surgery recovered to good facial function (H&B 1–2) at the last follow-up. In those aged 41–50, the rate of facial recovery was 76%; in those 51–60, it was 57%; and in those over 60, it was 80%. Treatment complications were rare and mainly classified as CDC [24] class 2 (i.e., medically treated vasospasm, venous thrombosis, or brain-edema), CDC 3a (i.e., nonsurgically treated CSF-fistula), or CDC 3b (i.e., surgically treated CSF fistulas, hemorrhages, hygroma, pneumocephalus, or hydrocephalus) (see Table 1).

In the present study cohort of large VS, the overall incidence of recurrence was 9%. The incidence of recurrence after respective monotherapy was significantly higher in *SRS* with 14% compared to *SURGERY* with 3% (see Fig. 1B). The incidence of recurrence of cystic VS (T3–T4) was 11%. Mean follow-up time was 79 (± 52.6) months in the whole study cohort, with 74 (± 52.7) months in *SURGERY* and 82 (± 52.2) months in *SRS*. Mean time to recurrence was longer in *SURGERY* with 102 (± 35.9) months compared to 57 (± 36.3) months in SRS (*p* = *0.007*).

**Fig. 2:**
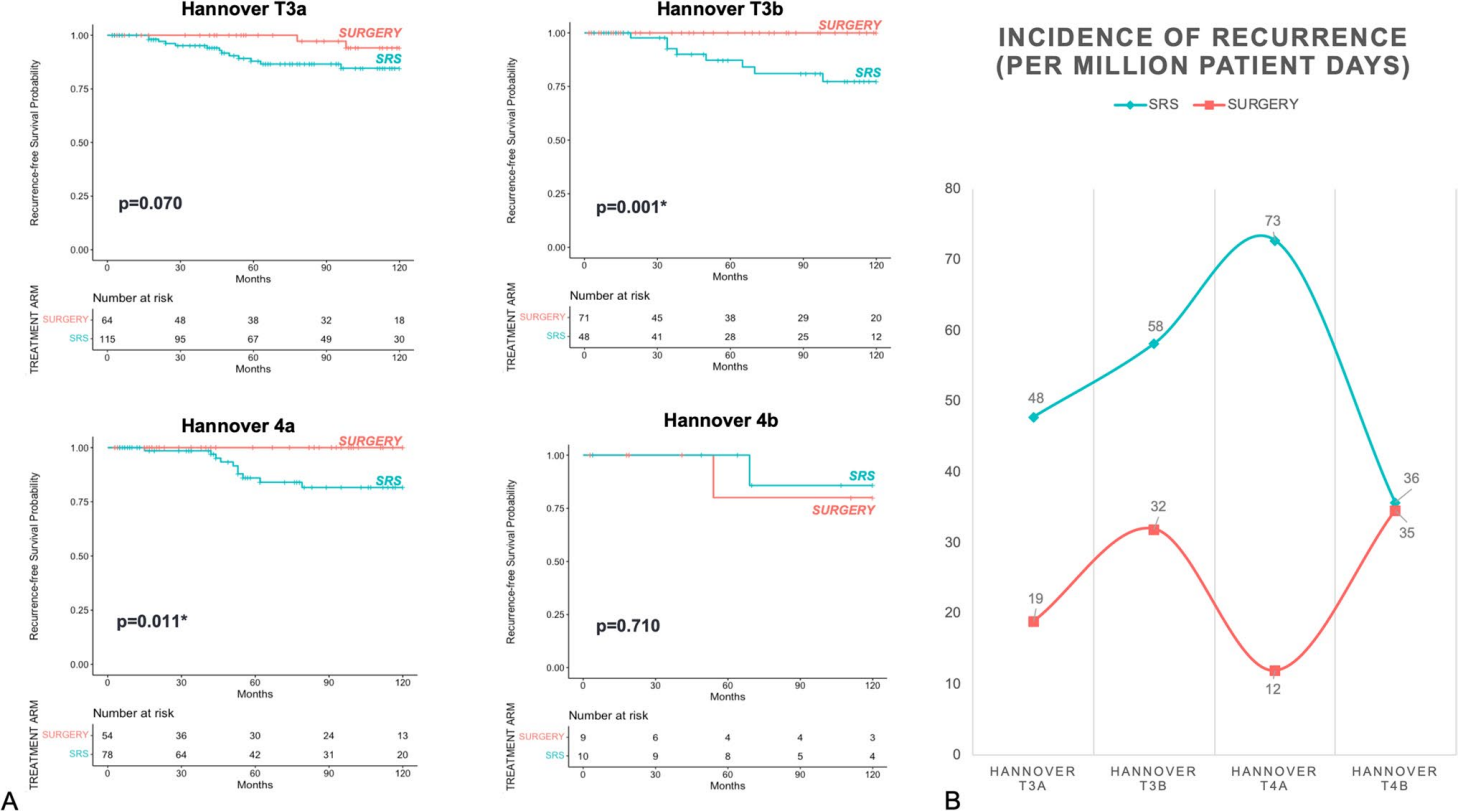
**A** 10-Year Kaplan–Meier-Analysis for tumor-recurrence in different tumor sizes (Hannover T3a-T4b) in *SRS* versus *SURGERY*. **B** shows incidence per patient time according to tumor size

Tumor size affected tumor control after both treatment measures (*SURGERY* and *SRS)* (Fig. 2A). In line, the incidence of recurrence per one million person days was higher in *SRS* compared to *SURGERY* and depended on tumor size (Fig. 2B). *SURGERY* was able to reduce events of recurrence by 42 events per one million patient days (*SRS*: 55 recurrences per one million patient days versus *SURGERY*: 13 recurrences per one million patient days) (Table 2). In patients treated with *SRS*, the rate of recurrence was the highest in patients over 40 years of age with 20% compared to older patient subgroups (41–50 years = 11%; 51–60 years = 13%; and > 60 years = 15%).

**Table 2:** Incidence of recurrence per patient time (per one million patient days), Number needed to operate, Number needed to harm, and Likelihood of Harm by patient and tumor characteristics

	*No. of Recurrences in SRS*	*Total person-days*	*No. of FFD in SRS*	*No. of Recurrences in SURGERY*	*Total person-days*	*No. of FFD in SURGERY*	*Incidence of recurrences (per million person-days) in SRS*	*Incidence of recurrences (per million person-days) in SURGERY*	*NNO*	*ARR (95CI) (%)*	*NNH*	*ARR (95CI) (%)*	*LLH (NNH/NNO)*
*Overall*	35/251 (14)	633.456	1/251 (0)	6/198 (3)	445.985	18/18 (9)	55	13	10	10.9% (6.0%-15.8%)	12	8.7% (4.6%-12.8%)	1.20
*Gender*													
*Male*	14/108 (13)	285.787	0/108 (0)	3/94 (3)	237.011	6/94 (6)	49	13	11	9.8% (2.5%-17.0%)	16	6.4% (1.4%-11.3%)	1.44
*Female*	21/143 (15)	347.969	1/143 (1)	3/104 (3)	208.974	12/143 (8)	60	14	9	11.8% (5.2%-18.4%)	13	7.7% (2.9%-12.4%)	1.45
*Age Groups*													
< *40 years*	3.15 (20)	40.026	0/15 (0)	0/58 (0)	93.297	5/58 (9)	75	0	5	20.0% (-0.2%;40.2%)	12	8.6% (1.4%;15.8%)	2.40
*40–50 years*	6/53 (11)	130.727	0/53 (0)	2/62 (3)	172.840	6/62 (10)	46	12	9	11.3% (2.8%-19.9%)	11	9.7% (2.3%;17.0%)	1.22
*50–60 years*	9/69 (13)	180.801	1/69 (1)	2/43 (5)	87.267	4/43 (9)	50	23	12	8.4% (-1.7%;18.5%)	13	7.9% (-1.3%;16.9%)	1.08
> *60 years*	17/114 (15)	282.202	0/114 (0)	2/35 (6)	92.581	3/35 (9)	60	22	11	9.2% (-0.9%;19.3%)	12	8.6 (-0.7%;17.9%)	1.09
*Tumor size*													
*Hannover T3a*	14/155 (13)	292.876	0/115 (0)	2/64 (3)	155.087	3/64 (5)	48	13	12	9.1% (1.7%;16.4%)	22	4.7% (-0.5%;9.9%)	1.83
*Hannover T3b*	11/78 (14)	189.186	0/78 (0)	2/54 (4)	117.736	3/54 (6)	58	17	10	10.4% (1.2%;19.6%)	18	5.6% (-0.6%;11.7%)	1.80
*Hannover T4a*	9/48 (19)	123.678	1/48 (0)	1/71 (1)	152.405	10/71 (14)	73	7	6	17.3% (5.9%;28.7%)	9	12.0% (2.9%;21.1%)	1.50
*Hannover T4b*	1/10 (10)	28.8016	0/10 (0)	1/10 (0)	20.757	2/10 (20)	36	48	N/A^*^	N/A	N/A	N/A	N/A
*Morphology*													
*Solid*	31/240 (13)	602.610	1/240 (1)	6/173 (3)	388.332	16/173 (9)	51	15	11	9.5% (4.4%-14.5%)	12	8.8% (4.4%-13.2%)	1.09
*Cystic*	4/11 (36)	31.146	0/11 (0)	0/25 (0)	57.653	2/25 (8)	128	0	3	36.4% (7.9%-64.8%)	13	8.0% (-2.6%-18.6%)	4.33

*NNO* number needed to operate, *ARR* absolute risk reduction, *ARI* absolute risk increase, *NNH* number needed to harm, *LHH* likelihood of harm

^*^NNO was calculated compared to overall SRS cohort

^**^NNO is negative

**Fig. 3:**
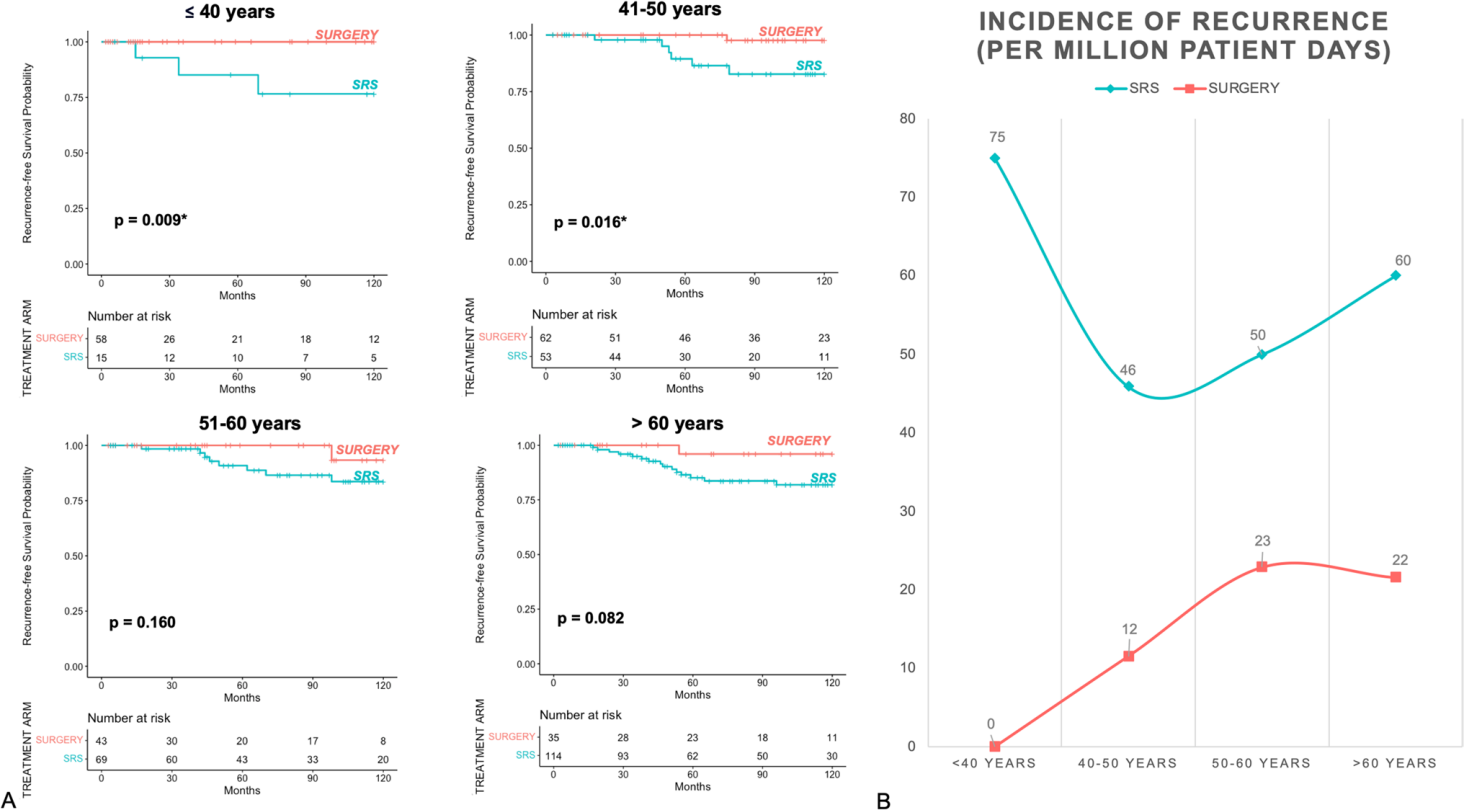
**A** shows the 10-Year Kaplan–Meier-Analysis for tumor-recurrence in different age groups: < 40 years, 40–50 years, 50–60 years and > 60 years in *SRS* versus *SURGERY*. **B** shows the incidence of recurrence per million patient days according to patient age

Comparative Kaplan–Meier analyses for *SURGERY* versus *SRS* depending on age are shown in Fig. 3A. In the *SRS*–treated group, the incidence of recurrences per patient time was the highest in those under 40 years old with 75 recurrences per 1 million patient-days and lowest in those aged 50–60 with 27. In the *SURGERY* group, tumor control illustrated per patient time was lower in the younger age groups (under 40 and 40–50) and higher in patients older then 50 and the elderly (Fig. 3B and Table 2).

**Fig. 4:**
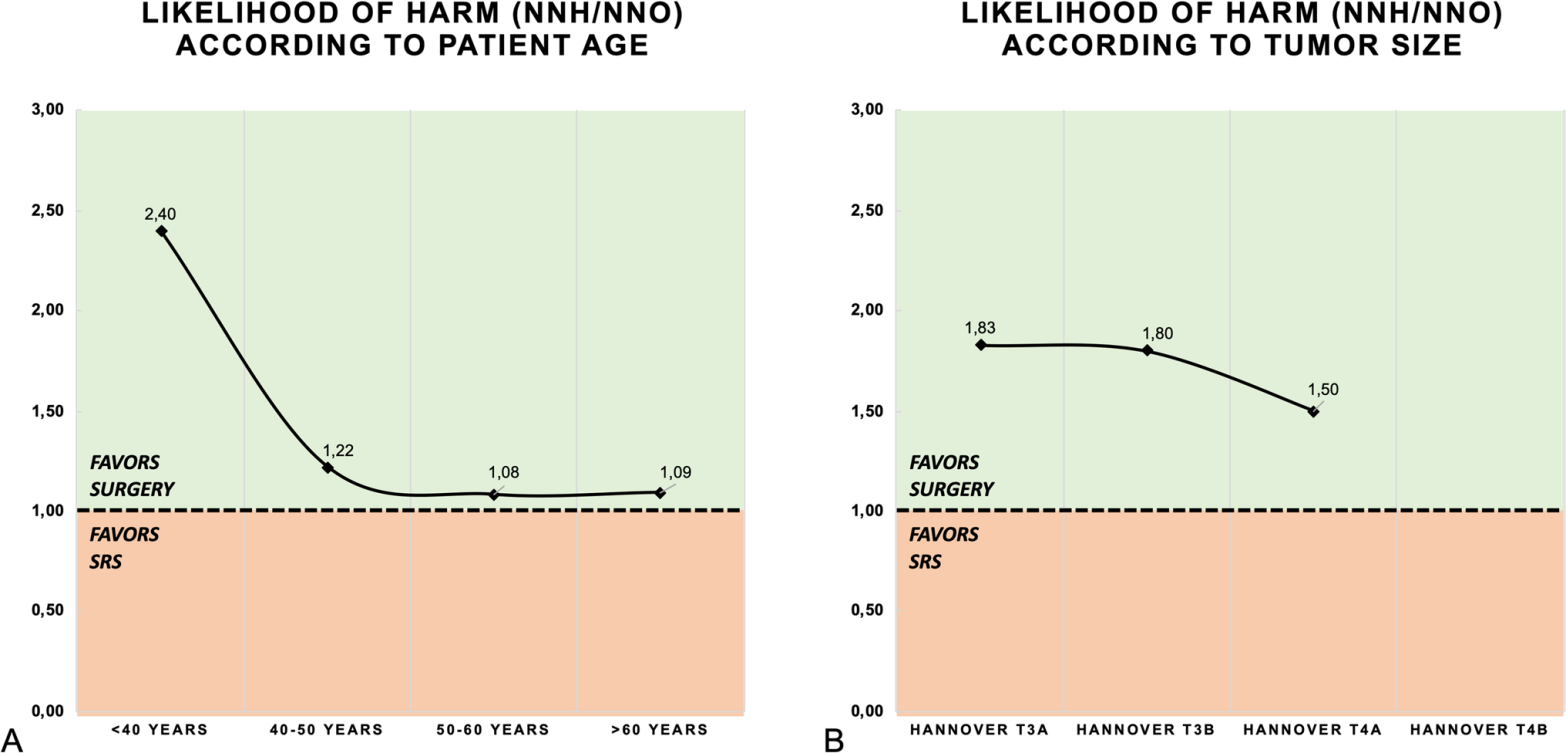
Likelihood of harm according to patient age and tumor size is shown in **A** and **B** respectively

The expression of difference in success/harm of both treatment arms are calculated and shown in Table 2. In the overall cohort, *SRS* presented with an incidence of recurrence of 14%, while *SURGERY* had a recurrence rate of 3%. Therefore, ARR was 11% (95%CI:6.0–15.8%), when treating patients with *SURGERY* instead of *SRS*. This yielded in a NNO of N = 10 (95CI: 6.3–16.6) – meaning that by treating 10 patients with *SURGERY* instead of *SRS*, one event of recurrence can be avoided. However, *SURGERY* increased the risk for FFD for 9% (95%CI:54.6%-12.7%) in *SURGERY* compared to *SRS*. FFD expressed by NNH was N = 12 (95CI:7.8–21.7). The overall LHH was therefore at 1.20. LHH was in favor of *SURGERY* in the following subgroups T3-T4a tumors, < 40 years of age and cystic VS (Table 2 and Fig. 4). The number of patients with T4b tumors were N = 10 in either group with each the same number of tumor recurrence (N = 1), and therefore the LLH-analysis was not applicable (Fig. 4).

## Discussion

Our study used data from a retrospective, dual–center study to report NNO/NNH depending on tumor and patients’ characteristics comparing *SURGERY* to *SRS* in large VS. In large VS (Hannover T3–T4), *SURGERY* was superior to *SRS*, considering tumor control, with an absolute risk reduction of 11% for the incidence of recurrence, resulting in an NNO of N = 10. In other words, one tumor recurrence can be prevented when N = 10 patients are treated by *SURGERY* instead of *SRS*. However, the absolute risk increase in FFD was 9% for *SURGERY* compared to *SRS*, yielding an NNH of N = 12. LLH was therefore 1.2 formally favoring *SURGERY* in large VS. LHH calculations indicated a benefit of *SURGERY* in T3 tumors, cystic VS, and young patients.

### Age–related differences and incidences per patient-time

Mean patient age was significantly higher in *SRS* subgroup indicating a provided-care bias towards more conservative management in the older patient cohort – an effect often seen in comparative studies [25, 26]. After all, *SRS* is a less invasive treatment option with less treatment-related side effects compared to *SURGERY* [6]. Nevertheless, *SURGERY* in the elderly was previously shown to be safe and the postoperative functional results to be similar in the elderly compared to the young, even though premorbid status was worse [27–29].

The most remarkable age trend was observed when we analyzed the incidence of tumor progression after *SRS* as a monotherapy: Here, the incidence of progression rose from 11–12% to 20% in patients under the age of 40. This result was also reflected in the incidence per patient-days (74.95 events per 1 million patient-days). In the Kaplan–Meier analysis, *SURGERY* was superior to *SRS* in patients under the age of 50. A retrospective, multicenter study with 176 patients showed a 5–year progression–free survival rate of 90.9% and a 10–year progression–free survival rate of 86.7% with single–session *SRS* in patients under the age of 45 with large VS (Koos III–IV). However, the basis for these calculations was data with a median follow-up of 3 years [30]. In the interpretation of tumor–control data, special attention has to be paid to the follow-up time because mean time-to-recurrence has been reported to be longer than 5 years, and shorter follow-up time may overestimate tumor control [5]. The strength of our work lies in its long follow-up period of 79 months in mean; therefore, a cumulative follow-up time of 1′112′639 patient-days (36.338 patient-months).

There are many ways to demonstrate tumor control as an endpoint in retrospective studies, including the following: incidence of recurrence, Kaplan–Meier analysis, and 5–year risk of recurrence, among others [30]. However, these measurements fail to express differences in saved recurrence/progression-free patient time and over- (Kaplan–Meier) or underestimate (5–year risk of recurrence) the true incidence of risk. By calculating the incidence per patient time, one is not only able to demonstrate the patient-days saved but also show the recurrence/progression rate in the context of the time to surveillance in the subgroups because this may vary in different age groups due to non–VS–related drop-outs or deaths.

### Risk–benefit-calculation

To the best of our knowledge, this is the first study to introduce the notion of NNO, NNH and LHH in neuro-oncological analysis. This model of analysis is highly reproducible and can be applied to any setting when assessment of treatment strategies is required and when balancing between the magnitude of the survival/recurrence advantage and side-effects is the goal [11]. LHH indeed has a strong visual impact, especially in the subgroup analysis of patient age and tumor sizes as shown in our analysis. LHH calculations in the present cohort indicated a benefit of *SURGERY* in T3 tumors, cystic VS and young patients.

Our study reported a low rate of relevant permanent facial palsy (9% in large VS) compared to that in the available literature, wherein this value varied largely between 14 and 66% [25, 26, 31–36]. We consider one of the strengths of this study that both centers were highly specialized in the treatment of VS with very high caseloads, so we were not comparing among different surgical techniques but truly intermodally between specialized *SRS* and *SURGERY* monotherapy [5]. Independent of the absolute values, the present analysis demonstrates—in contrast to the simplified perception in current literature [1]—that the risk–benefit analysis in VS does not unequivocally favor *SRS*. If we assume that tumor recurrence and FFD are equivalent in importance for patients’ onco-functional outcome, *SURGERY*—in this case, GTR—is justifiable even in light of the additional risk for FFD. Moreover, we demonstrated how NNO, NNH, and LHH varied depending on patients’ characteristics such as age, sex, tumor size, and tumor morphology. Our findings quantify the benefits of prioritizing SURGERY in large VS, particularly in T3a, T3b, T4a, cystic VS, and young VS patients.

Also, this study is intentionally designed to be thought-provoking: FFD versus tumor control are drastically placed side by side in this comparative methodology placing both aspects at equal importance. We deliberately chose to be radical in only including patients treated with GTR to compare the most aggressive surgical therapy (and therefore the treatment with the supposedly highest rate of facial morbidity) with the least-invasive and most functionally preservative treatment (*SRS*) to emphasize the analysis endpoints in both extremes (i.e. LLH) [1]. The fact that facial preservation rates vary largely between different academic neurosurgical centers, has resulted in a vivid discussion on intentional subtotal resection in VS to increase facial function preservation and therefore decrease risk of harm (NNH) [1, 25, 26, 31–36]. However, it has been shown that tumor control worsens with increasing residual tumor, and therefore we would assume that NNO for tumor control would also increase in lower EOR grades [37–39]. Its proportional effect on LLH has to be evaluated in the future on different EOR grades and combination therapy to better illuminate the question on the impact of EOR in VS management.

### Strength and limitations of this study

This study is limited by its nature of retrospective design. Even though the number of patients in this study was rather large—especially compared to the existing literature on large VS— our analysis could be even more meaningful in larger epidemiological study groups. NNT or NNO is a measurement of overall effect sizes among a cohort, so its direct translation cannot be applied directly to an individual patient and affect an individual treatment decision [9]. However, because this study demonstrates how the measures change in different subgroups (e.g., tumor size, patients’ ages, etc.), these factors can influence in individual treatment choice and consultation.

## Conclusions

In this study, ARI for facial palsy and ARR for incidence of recurrence were comparable at 11% and 9%, respectively, and yielded an LHH of 1.2. Independent of the absolute values, the present analysis demonstrates that the risk–benefit analysis in large VS does not unequivocally favor *SRS*. Still, large VS should be treated only in specialized centers, which have enough experience to ensure a high rate of facial preservation in large VS.

### Supplementary Information

Below is the link to the electronic supplementary material.

Supplementary file1 (DOCX 14 KB)

## Data Availability

No datasets were generated or analysed during the current study.

## Abbreviations

ARI: Absolute risk increase
ARR: Absolute risk reduction
CCI: Charlson comorbidities index
CDC: Clavien–Dindo classification
CPA: Cerebellopontine angle
CTC: Clinical tumor control
DS: Decompression surgery
ENT: Ear, nose, and throat
EOR: Extent of resection
FFD: Facial–function deterioration
FU: Follow-up
GKR: Gamma–Knife radiosurgery
GR: Gardner–Robertson
GTR: Gross total resection
H&B: House–Brackman
IAC: Internal auditory canal
KPS: Karnofsky Performance Score
LHH: Likelihood of harm/help
MRI: Magnetic resonance imaging
N/A: Not applicable
NNH: Number needed to harm
NNO: Number needed to operate
NNT: Number needed to treat
RFS: Recurrence–free survival
RTC: Radiographic tumor control
SRS: Stereotactic radiosurgery
ST: Salvage therapy
STR: Subtotal resection
STROBE: Strengthening the Reporting of Observational Studies in Epidemiology
SURGERY: Microsurgical tumor resection
TV: Tumor volume
VS: Vestibular schwannoma
