# Supplementary material for: Likelihood-of-harm/help of microsurgery compared to radiosurgery in large vestibular schwannoma
Source: J Neurooncol. 2024 Jun 29;169(2):299–308. doi: 10.1007/s11060-024-04732-0 (PMC11341602; doi:10.1007/s11060-024-04732-0)
Supplement: Supplementary file 1 — Supplementary file1 (DOCX 14 KB) [file 11060_2024_4732_MOESM1_ESM.docx]

**Likelihood-of-Harm/Help of Microsurgery Compared to Radiosurgery in Large Vestibular Schwannoma**

Sophie Shih-Yüng Wang, MD^1^; Gerhard Horstmann, MD^2^, Albertus van Eck, MD^2^; Marcos Tatagiba, MD^1^; Georgios Naros, MD^1^

^1^ Department of Neurosurgery, Eberhard Karls University, Tubingen, Germany

^2^ Gamma Knife Center, Krefeld, Germany

**SUPPLEMENTARY MATERIALS**

**Subtotal Resection**

The rate of STR was 5.26% (N=11/209) overall. The STR cohort consisted of 9% (N=1/11) Hannover T3a VS, 46% (N=5/11) Hannover T3b VS, 27% (N=3/11) Hannover T4a VS and 18% (N=2/11) Hannover T4b VS. The rate of tumor progression after STR was 36% (N=4/11) with a mean time to recurrence of 96 months (±32.09) months, which is significantly longer compared to SRS (p<.*0001*). The total number of follow-up person-days was 32'898 yielding in an incidence of recurrence of 121.59 events per million person-days, which is relevantly higher compared to GTR at 13 events per million person-days and 55 events per million person-days in *SRS*. Long-term facial function deterioration was 27% (N=3/11).

This yielded in an ARI of 22.42% (95CI:-6.33%-51.17%) for STR compared to *SRS* (both monotherapy), yielding in a NNH of N=5 considering tumor. When comparing treatment effectiveness in tumor control between STR and GTR, STR was also inferior with an ARI of 33.33% (95CI:4.81%-61.86%) and a NNH of N=3. However, the statistical significance and importance of this analysis has to be put into account to the very small number of STR, which was exactly the reason, why this subgroup was chosen to be excluded from the analysis.
